# The effects of the introduction of a chronic care model-based program on utilization of healthcare resources: the results of the Puglia care program

**DOI:** 10.1186/s12913-018-3075-0

**Published:** 2018-05-25

**Authors:** Fabio Robusto, Lucia Bisceglia, Vito Petrarolo, Francesca Avolio, Elisabetta Graps, Ettore Attolini, Eleonora Nacchiero, Vito Lepore

**Affiliations:** 1 Regional Healthcare Agency of Puglia Region (AReSS Puglia), via Giovanni Gentile n 52 -, 70126 Bari, Italy; 2 AReSS Puglia, Bari, Italy

**Keywords:** Chronic care model, Healthcare expenditure, Administrative databases, Electronic medical records, Unplanned hospitalizations, Drug costs, Out-patients, DDCI, stratification, frailty

## Abstract

**Background:**

Ageing is continuously increasing the prevalence of patients with chronic conditions, putting pressure on the sustainability of Healthcare Systems. Chronic Care Models (CCM) have been used to address the needs of frail people in the continuum of care, testifying to an improvement in health outcomes and more efficient access to healthcare services. The impact of CCM deployment has already been experienced in a selected cohort of patients affected by specific chronic illnesses. We have investigated its effects in a heterogeneous frail cohort included in a regional CCM-based program.

**Methods:**

a retrospective population-based cohort study was carried out involving a non-oncological cohort of adult subjects with chronic diseases included in the CCM-oriented program (Puglia Care). Individuals in usual care with comparable demographic and clinical characteristics were selected for matched pair analysis. Study cohorts were defined by using a record linkage analysis of administrative databases and electronic medical records, including data on the adult population in the 6 local area health authorities of Puglia in Italy (approximately 2 million people). The effects of Puglia Care on the utilizations of healthcare resources were evaluated both in a before-after and in a case-control analysis.

**Results:**

There were 1074 subjects included in Puglia Care and 2126 matched controls. In before-after analysis of the Puglia Care cohort, 240 unplanned hospitalizations occurred in the pre-inclusion period, while 239 were registered during follow-up. The incidence of unplanned hospitalization was 10.3 per 100 person/year (95% CI, 9.1–11.7) during follow-up and 12.1 per 100 person/year (95% CI, 10.7–13.8) in the pre-inclusion period (IRR, 0.84; 95% CI, 0.80–0.99). During follow-up a significant reduction in costs related to unplanned hospitalizations (IRR, 0.92; 95% CI, 0.91–0.92) was registered, while costs related to drugs (IRR, 1.14; *p* < 0.01), out-patient specialist visits (IRR, 1.19; *p* < 0.01), and planned hospitalization (IRR 1.03; p < 0.01) increased significantly. These modifications can be related to the aging of the population and modifications to healthcare delivery; for this reason, a case-control analysis was performed. The results testify to a significantly lower number (IRR, 0.79; 95% CI, 0.68–0.91), length of hospital stay (IRR, 0.80; 95% CI, 0.76–0.84), and costs related to unplanned hospitalizations (IRR, 0.80; 95% CI, 0.80–0.80) during follow-up in the intervention group. However, there was a higher increase in costs of hospitalizations, drugs and out-patients specialist visits during follow-up in Puglia Care when compared with patients in usual care.

**Conclusion:**

In a population-based cohort, inclusion of chronic patients in a CCM-based program was significantly associated with a lower recourse to unplanned hospital admissions when compared with patients in usual care with comparable clinical and demographic characteristics.

**Electronic supplementary material:**

The online version of this article (10.1186/s12913-018-3075-0) contains supplementary material, which is available to authorized users.

## Background

Aging of the population is continuously increasing the prevalence of chronic illnesses. Co-morbidity is associated with the worst health outcomes and increased healthcare expenditure [1, 2]. This is even more true in Healthcare Systems that show an acute and intramural oriented assistance [3]; in which fragmentation, poor coordination of care and lack of patient involvement results in inadequate responses to the needs of older people with chronic illnesses [4]. The inadequate management of patients with chronicity??chronic conditions is putting pressure on the sustainability of Healthcare Systems [5]. For this reason, the promotion of proactive, integrated, and personalized care is indispensable to optimize the use of healthcare resources [6, 7]. Chronic Care Models (CCM) have been used to address the needs of frail people in the continuum of care, testifying to an improvement in health outcomes and more efficient access to healthcare services for these patients [8]. The elements that characterize the CCM framework are: 1) an appropriately organized delivery system that contributes to producing interaction between patients and community healthcare resources; 2) supporting and improving of self-management [9]; 3) promoting interaction with patients, integrating guidelines with patient preferences [10]; 4) timely access to clinical data about patients through a computerized registry; 5) care coordination between healthcare teams [11]; 6) regular interaction between caregivers and patients, preferring a “continuous healing relationship” to face-to-face visits [12]. The deployment of a program of management of chronic patients at regional level requires the clinical risk stratification of the entire regional population and the identification of patients to be included in the CCM. The development of an infrastructure framework able to record patients’ clinical data in the continuum of care and the use of a widely applicable risk stratification tool are indispensable steps in this process [13].

The impact of CCM deployment on health care outcomes and the utilization of health care resources have already been trialled in a selected cohort of patients affected by specific chronic illnesses [14–18]. The deployment of an integrated framework based on a CCM have also testified to the improved quality of care and health outcomes in more heterogeneous cohorts of patients with chronic conditions [19]. In fact, the effect of the application of CCM on utilization and clinical outcomes had already been trialled in clinical and/or socially frail people [20–22], but a quantitative analysis on direct healthcare expenditure and the modality of recourse to the healthcare system had not previously been performed.

## Objectives

To evaluate the impact of the deployment of a CCM on direct healthcare expenditures and the modality of recourse to the healthcare system in a heterogeneous regional population.

## Methods

We conducted a dynamic retrospective population-based cohort study, using a record linkage analysis of administrative databases (ADs) and electronic medical records (EMRs), including data on the adult population (aged 40 years or over) in the 6 local area health authorities of Puglia in Italy (approximately 2 million people).

### Puglia care framework

**Fig. 1:**
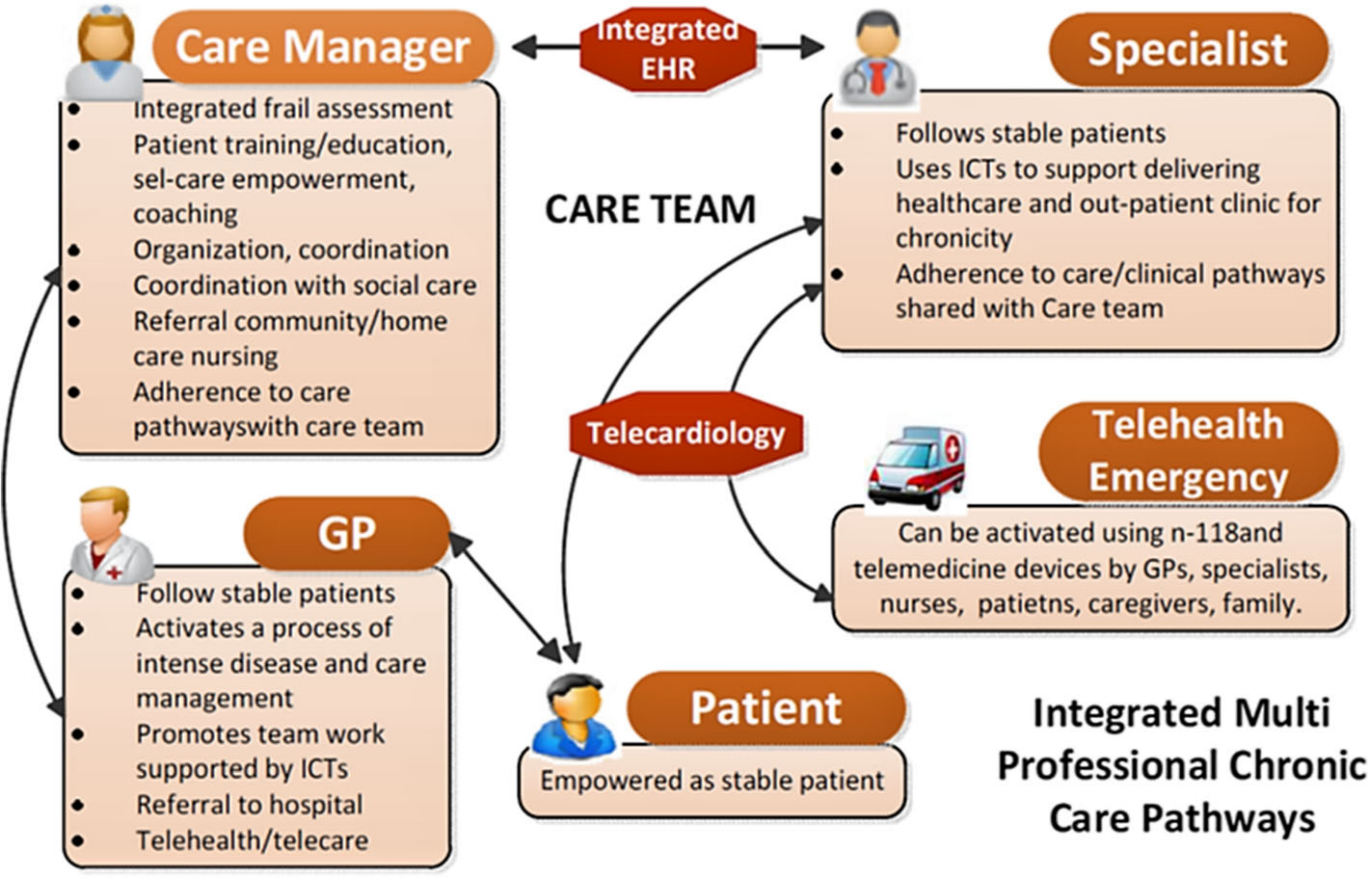
Puglia Care framework

The models and frameworks for Puglia Care development were derived from Wagner’s CCM [11]. The CCM was conceptualized from a primary care perspective and the improvement of its six interrelated components - self-management support, clinical information systems, delivery system redesign, decision support, health care organization, and community resources - advocate the production of better functional and clinical outcomes, resulting in Health care system reform [12]. The production of productive interaction between informed, activated patients and prepared, pro-active teams requires the implementation of a technological framework and the development of new professional figures [23]. To address the basic principles and elements of the CCM, Puglia Care introduced an information and communications technology system in which GPs, territorial specialists, and health professionals can access a web-based platform containing patient’s clinical, anthropometric, and socio-economic data. Moreover, on each patient’s electronic card it is possible to store the results of laboratory tests or imaging and measurement of telemedicine devices used in more complex patients to monitor glycaemia, saturation, blood pressure, and heart rate. A care manager was assigned to each patient included in Puglia Care. The care manager is involved in coordination of the communication process between the patient, their family and healthcare providers, monitoring and promotion of therapeutic compliance, coordination of healthcare with General Practitioners (GPs), and promotion of a more active and healthy lifestyle. Puglia Care promotes greater coordination among all healthcare managers and providers, and an immediate sharing of patient’s health documentation. Furthermore, the availability of health and social data and the presence of process and outcome variables in Puglia Care EMRs provide the policy maker with information about the effects of healthcare strategies (Fig. 1).

### Data sources

Specific registries collect data about hospitalizations, drug prescriptions and out-patient specialist visits which occurred in the Italian National Health System (NHS). In hospital discharge records, information about primary diagnosis, main co-morbility, and performed procedures are coded according to the International Classification of Diseases, Ninth Revision (ICD-9 CM) [24]. Out-Patients specialist visit databases provide information about the typology of territorial laboratory tests, imaging studies and out-patient specialist visits. In territorial prescription databases, all drugs reimbursed by the NHS are coded according to the Anatomical Therapeutic Chemical (ATC) classification system [25]. Costs related to hospitalizations, out-patient services and drug prescriptions are recorded in their specific registry. The civil registry provides information about age, sex, death, and migration. All security and protection measures for data from patients were performed according to the national law (Additional file 1).

### Intervention and control groups

A non-oncological cohort of subjects was selected from the EMRs of patients included in Puglia Care aged 40 years or above included in the intervention programs during the period from January 1, 2012 to December 31, 2013. For each patient, the date of inclusion in Puglia Care was considered as the index date. In parallel, we analyzed all the administrative data from the Puglia Region from 2011 to 2014. Administrative and EMR data were clustered for each patient included in Puglia Care through anonymous record linkage procedures. The period between the beginning of the observation period and the index date was considered as the pre-inclusion phase. Administrative data on the pre-inclusion period were used to define the baseline characteristics of the population. Patients were followed up from the index date to the date of death, migration, or end of the observation period (December 31, 2014). Subjects with less than 12 months of follow-up were excluded from the study.

Cluster analysis was used to include 2 subjects in usual care who presented the same clinic-demographic characteristic at index date and a minimum follow-up period of 12 months for each subject in the intervention group.

### Outcome variables

The main outcome was the average number of unplanned hospitalizations. Secondly, we also evaluated the average number of days of unplanned hospitalization and the total direct healthcare expenditure (costs of hospitalization, drugs, and outpatient specialist visits). We compared the primary and secondary outcome of patients included in Puglia Care with those of subjects in usual care in the follow-up period. Moreover, we also performed a before-after study in the cohort of patients included in Puglia Care, comparing outcomes in the pre-inclusion and follow-up periods.

### Statistical analysis

A matched pair 1-to-2 analysis was used to allow an unbiased comparison between patients included in Puglia Care and those in usual care [26, 27]. Patients in the control group were selected if at the index date they showed the same age, sex, healthcare district and number of unplanned hospitalization in the pre-inclusion period, and the same DDCI (Drug Derived Complexity Index) class [28] as patients included in Puglia Care. The characteristics of the study population and its subgroups are reported as percentages, means (standard deviation), or median (range). Incidence rates (IRs) with 95% confidence intervals of number and days of unplanned hospitalization and total direct healthcare expenditure per 100 person/years were estimated both in the two groups and in the pre-inclusion and follow-up periods in patients included in Puglia Care. Crude IR ratios (IRRs) were estimated using Poisson regression comparing patients included in Puglia Care with those in usual care. Risks were reported as IIRs along with their 95% confidence intervals and *P* values. All statistical analyses were performed using SAS version 9.4 (SAS Institute Inc).

## Results

Overall, 1112 non-oncological patients aged > = 40 years old were identified in Puglia Care from January 01, 2012 to December 31, 2013. All these subjects presented at least one follow-up control every 6 months. For each subject, EMR data was linked with those of regional administrative databases produced from January 1, 2011 to December 31, 2014, identifying 1074 subjects aged 40 years or above who were present both in EMRs and the regional civil registry with a minimum pre-inclusion and follow-up period of 12 months. In parallel, 2126 controls with the same clinic-demographical characteristics (age, sex, healthcare district, DDCI class, number of unplanned hospitalization in the pre-inclusion period) at inclusion data were identified. Pre-matching characteristics differed significantly between subjects included in Puglia Care and the population in usual care, but the matching gave a good balance for all the considered characteristics (Table 1). The two samples also presented a similar median pre-inclusion period (22.0 months in the Puglia Care group and 21.9 months in the control group) and a similar duration of follow-up (25.8 and 25.9 months, respectively).

**Table 1:** Study cohorts characteristics

Characteristic	Intervention group	Usual care
N°	1074	2126
Age		
Mean (Std)	66.2 (10.1)	66.2 (10.2)
Median (range)	66.4 (40.4–92.6)	66.6 (40.0–92.5)
Males (%)	490 (45.6)	968 (45.5)
Health care districts, N° (%)		
1	13 (1.2)	26 (1.2)
2	12 (1.1)	22 (1.0)
3	295 (27.5)	586 (27.6)
4	298 (27.8)	593 (27.9)
5	22 (2.1)	44 (2.1)
6	1 (0.1)	2 (0.1)
7	80 (7.5)	158 (7.4)
8	109 (10.1)	217 (10.2)
9	17 (1.6)	34 (1.6)
10	172 (16.0)	342 (16.1)
11	20 (1.9)	34 (1.6)
12	3 (0.3)	6 (0.3)
13	32 (3.0)	62 (2.9)
Local Health Authority, N° (%)		
160,106	80 (7.5)	158 (7.4)
160,112	20 (1.9)	34 (1.6)
160,113	298 (27.8)	593 (27.9)
160,114	29 (2.7)	56 (2.6)
160,115	32 (3.0)	62 (2.9)
160,116	615 (57.3)	1223 (57.3)
DDCI, N° (%)		
≤0	114 (10.6)	228 (10.7)
1–2	312 (29.1)	624 (29.3)
3–4	287 (26.7)	572 (26.9)
5–6	164 (15.3)	325 (15.3)
≥7	197 (18.3)	377 (17.7)
N° of unplanned hospitalization in pre-inclusion period		
N°	240	393
Mean (Std)	0.22 (0.69)	0.18 (0.53)
Median (range)	0 (0–8)	0 (0–4)

### Before-after analysis in the Puglia care group

During the pre-inclusion period, 240 unplanned hospitalizations (with a total of 1734 days of hospitalization) and 239 hospitalizations (with a total of 2147 days) occurred during follow-up in the Puglia Care group (Additional file 2: Table S1). The overall IR of total unplanned hospitalizations was 12.1 (95% CI, 10.7–13.8) per 100 person/year during the pre-inclusion period and 10.3 (95% CI, 9.1–11.7) during follow-up; while, the IR of total days of unplanned hospitalization were 87.8 (95% IC, 83.7–92.0) and 92.6 (88.8–96.6) respectively. During follow-up there was a significant reduction in the number of unplanned hospitalizations (IRR, 0.84; 95% CI, 0.80–0.99); while, a not statistically significant increase in days of unplanned hospitalizations was registered (IRR, 1.04; 95% CI, 0.98–1.11). Regarding direct healthcare costs, during follow-up a significant reduction in costs related to unplanned hospitalizations (IRR, 0.92; 95% CI, 0.91–0.92) was registered; whereas total costs for hospitalizations (IRR, 1.03; 95% CI, 1.03–1.03), drugs (IRR, 1.14; 95% CI, 1.14–1.14), and outpatients specialist visits (IRR 1.19; 95% CI, 1.19–1.19) increased significantly (Table 2).

**Table 2:** Incidence Rates of total number and days of unplanned hospitalizations and direct healthcare costs (in Euros) per 100 person/years and relative Incidence Rate Ratios in Care Puglia Group. Comparison pre-inclusion period and follow-up

Outcomes	Pre-inclusion (95% CI)	Follow-up (95% CI)	IRR (95% CI)
N° of unplanned hospitalizations	12.1 (10.7–13.8)	10.3 (9.1–11.7)	0.84 (0.80–0.99)*
Days of unplanned hospitalizations	87.8 (83.7–92.0)	92.6 (88.8–96.6)	1.04 (0.98–1.11)
Total cost of hospitalizations	86,189 (86060–86,319)	90,059 (89936–90,181)	1.03(1.03–1.03)*
Cost of unplanned hospitalizations	42,458 (42367–42,549)	39,572 (39491–39,653)	0.92 (0.91–0.92)*
Cost of drugs	81,641 (81515–81,767)	93,706 (93581–93,831)	1.14 (1.14–1.14)*
Cost of out-patient specialistic visits	32,783 (32704–32,863)	39,239 (39158–39,319)	1.19 (1.19–1.19)*

*p < 0.05

These modifications to healthcare expenditure could be related to the aging of the population and modifications to healthcare delivery. For this reason, we performed a case-control analysis with a cohort of subjects with similar clinical and demographical characteristics.

### Case control analysis

During the pre-inclusion period, 393 unplanned hospitalizations (for a total of 3169 days of hospitalization) and 672 hospitalizations (for a total of 5946 days) were registered during follow-up in the control group (Additional file 2: Table S1). The overall IR of total unplanned hospitalizations during the pre-inclusion period was 12.1 (95% CI, 10.7–13.8) per 100 persons/year in the intervention group and 11.7 (95% CI, 10.6–12.9) in the usual care group; while, the IR of total days of unplanned hospitalization were 87.8 (95% IC, 83.7–92.0) and 94.2 (91.0–97.6), respectively. To analyze direct healthcare expenditure, we evaluated the IRs of the total cost of hospitalizations, unplanned hospitalizations, drugs and outpatient specialist visits (the results are reported in Euros). While no statistical differences in the total number and days of unplanned hospitalizations were found between the two groups in the pre-inclusion period; expenditure for hospitalizations (IRR, 1.10; 95% CI, 1.10–1.11), unplanned hospitalization (IRR, 1.02; 95% CI, 1.01–1.02), and drugs (IRR, 1.20; 95% CI, 1.20–1.20) are statistically significantly higher in the Puglia Care group (Table 3).

**Table 3:** Incidence Rates of total number and days of unplanned hospitalizations and direct healthcare costs (in Euros) per 100 person/years and relative Incidence Rate Ratios in pre-inclusion period

Outcomes	Puglia Care (95% CI)	Usual care (95% CI)	IRR (95% CI)
N° unplanned hospitalizations	12.1 (10.7–13.8)	11.7 (10.6–12.9)	1.04 (0.88–1.22)
Days of unplanned hospitalizations	87.8 (83.7–92.0)	94.2 (91.0–97.6)	0.93 (0.88–1.00)
Total cost of hospitalizations	86,189 (86060–86,319)	77,962 (77868–78,056)	1.10 (1.10–1.11)*
Cost of unplanned hospitalizations	42,458 (42367–42,549)	41,548 (41480–41,617)	1.02 (1.01–1.02)*
Cost of drugs	81,641 (81515–81,767)	67,868 (67780–67,956)	1.20 (1.20–1.20)*
Cost of out-patient specialistic visits	32,783 (32704–32,863)	33,243 (33182–33,305)	0.99 (0.98–0.99)*

*p < 0.05

During the follow-up period, there was a significant reduction in the number of unplanned hospitalizations in the intervention group (IR, 10.3; 95% CI, 9.1–11.7) and a significant increase in the control group (IR, 13.1; 95% CI, 12.1–14.1). Regarding days of unplanned hospitalizations, although a rise of IRs was detected in both groups, this increase was higher in the usual care group, determining a significantly lower recourse to days of unplanned hospitalization in the Puglia Care group during follow-up (IRR, 0.80; CI, 0.76–0.84). The minor recourse to emergency services in the Puglia Care group during follow-up was also testified by the reduction in healthcare expenditure related to unplanned hospitalizations (IRR, 0.80; 95% CI, 0.80–0.80).

On the other hand, IRs of total costs of hospitalizations, drugs and out-patient specialist visits increased during follow-up in both groups, but with important differences. In fact, while the IRR of the overall cost of hospitalizations between the Puglia Care and control group was less during follow-up (IRR, 1.02; 95% CI 1.02–1.02) than during the pre-inclusion period (IRR, 1.10; 95% CI, 1.10–1.11), the increase in the cost of drugs (IRR 1.24, 95% CI, 1.24–1.24) and outpatient specialist visits (IRR, 1.07; 95% CI, 1.07–1.08) costs in the Puglia Care group became more evident during follow-up (Table 4).

**Table 4:** Incidence Rates of total number and days of unplanned hospitalizations and direct healthcare costs (in Euros) per 100 person/years and relative Incidence Rate Ratios during follow-up

Outcomes	Intervention group	Usual care group	IRR (CI)
N° of unplanned hospitalizations	10.3 (9.1–11.7)	13.1 (12.1–14.1)	0.79 (0.68–0.91)*
Days of unplanned hospitalizations	92.6 (88.8–96.6)	115.9 (113.0–118.9)	0.80 (0.76–0.84)*
Total cost of hospitalizations	90,059 (89936–90,181)	88,466 (88385–885,475)	1.02 (1.02–1.02)*
Cost of unplanned hospitalizations	39,572 (39491–39,653)	49,622 (49562–49,683)	0.80 (0.80–0.80)*
Cost of drugs	93,706 (93581–93,831)	75,369 (75294–75,444)	1.24 (1.24–1.24)*
Cost of out-patient specialistic visits	39,239 (39158–39,319)	36,492 (36440–36,545)	1.07 (1.07–1.08)*

*p < 0.05

## Discussion

Our study shows that the implementation of CCM for frail patients is associated with less recourse to and a significant reduction in costs due to unplanned hospitalizations. These findings suggest that integrated care may lead to a reduced utilization of the emergency system by patients with co-morbidity. The effectiveness of the CCM has been tested in a few trials, having mixed results regarding the improvement in process and outcomes of care [29–33]. Moreover, the great majority of these studies have analyzed the impact of CCMs in selected cohorts of patients, investigating outcomes relevant to specific chronic diseases (monitoring of diabetes, cardio-vascular risks, blood pressure, BMI,..) [34, 35]. Few randomized controlled trials have enrolled a nonspecific chronic disease cohort with multi-morbidity, and none have investigated the impact of CCM on modality of recourse to the health care system [36–38]. The results of retrospective cohort studies, as well as case studies and case series, offer a greater numerical sample and an analysis of impact on healthcare practice. The vast majority of these studies included diabetic patients, assessing the effectiveness of CCMs for improving health outcomes [39–41]; however, knowledge on CCM cost-effectiveness in diabetes care is nascent [42]. The only data available have testified that CCM reduced lifetime risks of blindness, end-stage renal disease, and coronary artery disease, in diabetic patients with a resulting increase in benefits in a cost range from $33,386/Quality Adjusted Life Years [43] to $42,051/Quality Adjusted Life Years [44].

A cost analysis on patients with chronic conditions was experimented in a large population of Tuscany Region through a Before-After study. This study showed that the application of activated diagnostic and therapeutic pathways, involving patients affected by the four most common chronic conditions (Diabetes Mellitus, Heart Failure, Chronic Obstructive Pulmonary Disease and stroke) reduces the recourse to hospitalization and A&E access, despite an increase in outpatient specialist visits and expenditure on drugs [16]. This study did not include a control group; for this reason it was not possible to determine whether modifications in recourse to hospitalization and A&E departments were caused by the disease management programs or by other factors. On the contrary, in our study we have analyzed the effects of CCM both in a before-after and in a control-group analysis, also evaluating modifications in access and costs of healthcare delivery in the control group. Moreover, a key strength of this study is the utilization of data for all patients of a regional healthcare system and the utilization of a matched pair analysis using demographic and clinical variables to match patients included in Puglia Care and subjects in a control group. These elements have allowed us to preserve a recruitment bias and compare outcomes between the two groups in a homogeneous case-mix population.

In the pre-inclusion period, the only significant differences between cases and controls were direct healthcare expenditure. In particular, data showed higher costs related to hospitalizations and drug consumption in the case group. This finding might not be related to differences in the case-mix of the population between the two groups, but rather it can be explained by the inclusion of patients in the control group who were low-users of healthcare resources with respect to patients with similar clinical characteristics included in the case group. In fact, GPs were called to select cases - including in this way patients who had regular contact with the healthcare service - while the selection of controls was random, also including low-compliant patients.

Although a greater increase in drugs costs and outpatients specialist visits was recorded during follow-up in the Puglia Care group compared with the control group, total expenditure due to unplanned hospitalizations registered an important reduction in the Puglia Care group during follow-up. The decrease in unplanned hospital admissions is only a part of the cost saving due to the reduction in the recourse to the emergency healthcare system; the overall impact of CCM on direct or indirect healthcare expenditure needs further studies, including the analysis of additional data.

### Limitations

The use of administrative data implies that the current study gives no information about some important quality indicators only available via medical records (e.g. smoking, weight control and blood pressure) [45]. Furthermore, the management programs based on the CCM described in our study were developed in a healthcare system with universal delivery; thus its effectiveness in commercial disease-management programs should be further investigated.

## Conclusions

Aging and the increased prevalence of chronic conditions are putting pressure on the sustainability of healthcare services. Modifications are indispensable to tailor healthcare delivery to patients’ needs, reducing the inappropriate recourse to emergency healthcare services and the consequent increase in care costs. The evaluation of the cost-effectiveness impact of alternative healthcare intervention is mandatory and nowadays information about the impact of CCMs on the healthcare pathways of chronic patients is emerging. Our study provides evidence documenting a reduction in days and costs of unplanned admissions in the CCM group when compared with subjects with similar demographic and clinical characteristics but persisting in usual care. On the other hand, the introduction of Puglia Care has produced and increased cost of drugs and out-patient visits; these costs are probably related to the definition of multi-specialist pathways of care and more appropriate pharmacological therapies. In conclusion, the deployment of a CCM-oriented strategy in frail subjects has proved its efficacy in the reduction of recourse to the emergency healthcare system, allowing a more appropriate redistribution of economic resources.

## Additional files

Additional file 1: Puglia_care.txt (database). (TXT 558 kb)

Additional file 2: **Table S1.** *Number, mean(std) and median(range) of end-points in pre-inclusion and follow-up. (DOCX 16 kb)*

## Abbreviations

ADs: Administrative databases
CCM: Chronic care model
DDCI: Drug derived complexity index
EMRs: Electronic medical records
GPs: General practitioners
IR: Incidence ratio
IRR: Incidence rate ratio
