# Supplementary material for: The effects of the introduction of a chronic care model-based program on utilization of healthcare resources: the results of the Puglia care program
Source: BMC Health Serv Res. 2018 May 25;18:377. doi: 10.1186/s12913-018-3075-0 (PMC5970509; doi:10.1186/s12913-018-3075-0)
Supplement: Supplementary file 2 — Table S1. Number, mean(std) and median(range) of end-points in pre-inclusion and follow-up. (DOCX 16 kb) [file 12913_2018_3075_MOESM2_ESM.docx]

***Additional file 2: Table S1. Number, mean(std) and median(range) of end-points in pre-inclusion and follow-up.***

| **Characteristic** | **Puglia Care** | **Usual care** |
| --- | --- | --- |
| **Unplanned hospitalization in pre-inclusion period**  N°  Mean (Std)  Median (range) | 240  0.22 (0.69)  0 (0-8) | 393  0.18 (0.53)  0 (0-4) |
| **Days of unplanned hospitalization in pre-inclusion period**  N°  Mean (Std)  Median (range) | 1734  1.61 (5.88)  0 (0-78) | 3169  1.49 (6.34)  0 (0-193) |
| **Total cost of hospitalization in pre-inclusion period**  N°  Mean (Std)  Median (range) | 1,702,938  1,586 (4,684)  0 (0-48,279) | 2,622,233  1,233 (3,723)  0 (0-61,467) |
| **Cost of unplanned hospitalization in pre-inclusion period**  N°  Mean (Std)  Median (range) | 838,900  781 (2,852)  0 (0-29,874) | 1,397,478  657 (2,486)  0 (0-61,467) |
| **Cost of drugs in pre-inclusion period**  N°  Mean (Std)  Median (range) | 1,613,085  1,502 (1,774)  997 (0-27,083) | 2,282,738  1,074 (1,334)  682 (0-21,266) |
| **Cost of outpatient specialistic visit in pre-inclusion period**  N°  Mean (Std)  Median (range) | 647,743  603 (710)  416 (0-8,942) | 1,118,143  526 (2,222)  243 (0-51,923) |
| **N° of unplanned hospitalization during follow-up**  N°  Mean (Std)  Median (range) | 239  0.22 (0.65)  0 (0-6) | 672  0.32 (0.84)  0 (0-8) |
| **Days of unplanned hospitalization during follow-up**  N°  Mean (Std)  Median (range) | 2147  2.00 (7.1)  0 (0-89) | 5946  2.80 (9.68)  0 (0-107) |
| **Total cost of hospitalization during follow-up**  N°  Mean (Std)  Median (range) | 2,086,871  1,943 (5,213)  0 (0-49,829) | 4,537,400  2,134 (5,811)  0 (0-62,010) |
| **Cost of unplanned hospitalization during follow-up**  N°  Mean (Std)  Median (range) | 916,978  854 (3,151)  0 (0-49,829) | 2,545,129  1,197 (4,036)  0 (0-44,321) |
| **Cost of drugs during follow-up**  N°  Mean (Std)  Median (range) | 2,171,387  2,022 (2,331)  1,456 (0-49,664) | 3,865,653  1,818 (2,499)  1,101 (0-35,764) |
| **Cost of outpatient specialistic visit during follow-up**  N°  Mean (Std)  Median (range) | 909,256  847 (1,173)  591 (0-21,971) | 1,871,688  880 (4,253)  371 (0-113,071) |
